# Enhanced von Neumann Entropy in Dense Two-Level Ensembles

**DOI:** 10.3390/e28101146

**Published:** 2026-10-18

**Authors:** Aurelian Isar, Tatiana Mihaescu, Mihai A. Macovei

**Affiliations:** 1 National Institute of Physics and Nuclear Engineering, 30 Reactorului, POB-MG6, RO-077125 Magurele, Romania; mihaescu.tatiana@theory.nipne.ro; 2 Institute of Applied Physics, Moldova State University, Academiei Str. 5, MD-2028 Chişinău, Moldova; mihai.macovei@ifa.usm.md

**Keywords:** von Neumann entropy, cooperative effects, dipole–dipole interactions

## Abstract

We investigate the equilibrium properties of the von Neumann entropy in a dense, dipole–dipole coupled ensemble of two-level emitters confined in a fully symmetric Dicke subspace, which additionally interact through a common thermal bath. The static dipole–dipole interactions are assumed to be strong, though remaining smaller than the transition frequency. Consequently, the resulting thermal equilibrium entropy of the ensemble is characterized as a function of these coupling strengths. As a consequence, we have demonstrated that the von Neumann entropy enhances when stronger dipole–dipole couplings among the two-level emitters are being involved. Furthermore, the entropy can even be reduced for an opposite sign of the dipole–dipole coupling strength compared to the former case.

## 1. Introduction

An atomic ensemble provides one of the most versatile settings for investigating and controlling the light–matter interaction interfaces in modern quantum technology [1,2]. Their distinctive feature is the collective radiative phenomena that arise in ensembles of closely spaced quantum emitters, such that the spatial extent of an ensemble is comparable to, or smaller than, the relevant radiation wavelength [3,4,5,6,7,8,9]. Subsequent extensions of these models include incoherently driven atomic ensembles, artificial atoms or coupled bosonic modes, interacting through common environmental modes, where stationary correlations and nonclassical radiation can arise even without coherent excitation [8,9,10,11,12]. Recent studies considered dense samples [13,14,15] and their open-system evolution [16,17], showing that the strong dipole–dipole interaction may alter the collective spectrum and modify the effective transition frequencies, equilibrium populations, and photon statistics, respectively.

Early studies of atom–field models established that the reduced von Neumann entropy of the atom is a useful, sensitive measure of quantum information exchange between a two-level atom and a laser field [18]. Moreover, von Neumann entropy was shown to capture changes in the reduced atomic state produced by intrinsic decoherence, arising from deviations from perfectly continuous unitary evolution [19]. Its significance extends across both quantum information theory and quantum thermodynamics, providing a basis-independent measure of the uncertainty associated with a quantum state. In quantum information, the von Neumann entropy measures mixedness and has a fundamental operational role, while for a globally pure bipartite state it becomes the entropy of entanglement of either subsystem [20,21]. In quantum thermodynamics, the von Neumann entropy is a fundamental quantity connecting the statistical description of quantum states with thermalization and the irreversibility of thermodynamic processes [22,23].

The von Neumann entropy became particularly relevant when considering the system of a two-level atom interacting with a finite-temperature electromagnetic bath, where the dissipative environment actively destroys phase information through decoherence and the entropy of the atom evolves dynamically from a pure quantum state into a mixed thermal equilibrium [24]. Similarly, thermal cavity fields have been considered explicitly in connection with entropy exchange in the Jaynes–Cummings model, capturing the effect of coherence loss [25]. Studies involving squeezed and structured reservoirs have shown that the atomic entropy is also sensitive to the statistical and spectral properties of the surrounding field, accelerating the loss of coherence and relaxation [26,27]. The dissipative cavity losses likewise modify the entropy and the purity of multi-level superpositions in atomic subsystems, setting the stage for studying systems with many degrees of freedom [28,29].

When considering systems composed of multiple emitters, the interpretation of von Neumann entropy becomes more subtle, since it coexists with quantum correlations developed between atoms, though they do not generally display identical dynamics [30,31,32,33]. In collective atomic systems described by Dicke-type models [15,17,34,35,36], and in multi-level atom–field configurations [37], the von Neumann entropy directly quantifies the entanglement between the collective atomic state and the electromagnetic field, considering that the global state is pure, while the collective coupling simultaneously generates entanglement among the atoms [34,35].

The setup becomes richer once interactions between the emitters and collective dissipation into a common environment are both present [15,38]. Unlike in a single emitter dissipation, two or more close-enough atoms interacting with a common bath may acquire correlations and stationary coherences in the system, as the two atoms appear to be indistinguishable to the environment [39]. Thus, the bath-induced coherences bring fundamental alterations to the energetic and entropic properties of two-level systems, creating the steady-state correlations [40]. More specifically, the thermodynamic description of the collectively dissipative systems relates the von Neumann entropy and quantum correlations to the thermodynamic quantities, such as internal energy and maximum extractable work [41,42]; see also Refs. [43,44,45,46,47].

Considering dense atomic samples, which enable stronger dipole–dipole coupling in a thermal electromagnetic field reservoir, one reaches a higher complexity problem with more fertile ground for interesting effects [7,8,9,10,13,14,15,16,38,42,48]. Direct dipole–dipole coupling has been shown to modify atomic mixedness and other quantum information quantities in decohering two-atom systems, resulting in the production and protection of coherence against thermal noise [33,49]. Additionally, strong dipole interaction has been shown to modify quantum information and metrological properties in decohering two-atom systems [50]. These results indicate that the entropy of an interacting atomic ensemble can provide a sensitive indicator of collective effects and cannot, in general, be inferred from the behavior of independent thermalized emitters.

In dense atomic ensembles, the dipole–dipole interaction may reshape the collective dynamics, and consequently the established thermal state may change as well. This motivates a systematic study of how the von Neumann entropy of a dense ensemble of two-level emitters depends on interatomic coupling, temperature, and ensemble size. Therefore, here, we investigate the quantum dynamics of a small, strongly dipole–dipole interacting two-level atomic ensemble in thermal equilibrium with its surrounding thermostat. We focus on the features of the von Neumann entropy when the dipole–dipole interaction strength is comparable to, but smaller than, the transition frequency of a single emitter. To this end, we present the corresponding master equation for this Dicke-like multi-qubit ensemble and its analytical steady-state equilibrium solution, respectively. Particularly, we have demonstrated that the von Neumann entropy enhances or suppresses when stronger dipole–dipole couplings among the two-level emitters are being considered, depending on their signs.

This paper is organized as follows. Section 2 outlines the theoretical framework and the system under consideration. Section 3 then details the features of the von Neumann entropy in dense atomic ensembles, analyzing the main findings. Concluding remarks and the summary are given in Section 4.

## 2. Theoretical Framework

We investigate an ensemble of *N* motionless and dipole–dipole coupled two-level emitters positioned at ${\vec{r}}_{j}$, j∈{1,2,⋯,N}, each with a transition frequency $\omega_{0}$ and interacting with a thermal reservoir at temperature *T*. The atomic subsystem is densely packed, meaning that its linear dimensions are much smaller than the photon emission wavelength λ, allowing us to operate in the Dicke limit [3,4,5,6]. Notice that thermal excitations are relevant when the emitter’s transition frequency lies within the microwave frequency domain, where the dipole–dipole interactions are strong as well. The superradiance effect, in these frequency ranges, was already experimentally demonstrated; see, e.g., [51]. Therefore, within the dipole and rotating-wave approximations [3,4,5,6,7,8,9], the Hamiltonian of the considered system is given by $H=H_{F}+H_{A}+H_{DD}+H_{AF}$, where

(1) $$\begin{array}{c} H_{F}=\sum_{k}\hbar \omega_{k}a_{k}^{†}a_{k}, \end{array}$$

is the free energy of the thermal electromagnetic field (EMF) reservoir, while

(2) $$\begin{array}{c} H_{A}=\sum_{j=1}^{N}\hbar \omega_{0}R_{zj}, \end{array}$$

is the corresponding free energy of the atomic subsystem. The next component of *H*, i.e.,

(3) $$\begin{array}{c} H_{DD}=-\hbar \sum_{j\neq l}\Omega_{jl}R_{j}^{+}R_{l}^{-}, \end{array}$$

describes the static dipole–dipole interactions within any two atoms separated by $r_{jl}=\left|{\vec{r}}_{j}-{\vec{r}}_{l}\right|$, while $\Omega_{jl}\propto r_{jl}^{-3}$. The weak interaction between the two subsystems is governed by the following Hamiltonian:

(4) $$\begin{array}{c} H_{AF}=i\sum_{k}\left({\vec{g}}_{k}\cdot \vec{d}\right)\left(a_{k}^{†}R^{-}-R^{+}a_{k}\right), \end{array}$$

where $\left({\vec{g}}_{k}\cdot \vec{d}\right)=\sqrt{2\pi \hbar \omega_{k}/V}\left({\vec{e}}_{p}\cdot \vec{d}\right)$ is the coupling strength between the emitters and the thermal EMF modes. Furthermore, ${\vec{e}}_{p}$ denotes the photon polarization vector with p∈{1,2}, and *V* represents the quantization volume. The collective atomic operators $R^{+}=\sum_{j=1}^{N}R_{j}^{+}=\sum_{j=1}^{N}{|e\rangle }_{\mathrm{jj}}\langle g|$ and $R^{-}={\left[R^{+}\right]}^{†}$ satisfy the standard commutation relations of the SU(2) algebra, namely $\left[R^{+},R^{-}\right]=2R_{z}$ and $\left[R_{z},R^{\pm }\right]=\pm R^{\pm }$. Here, $R_{z}=\sum_{j=1}^{N}R_{zj}=\sum_{j=1}^{N}\left({|e\rangle }_{\mathrm{jj}}\langle e|-{|g\rangle }_{\mathrm{jj}}\langle g|\right)/2$ represents the collective population inversion operator, where ${|e\rangle }_{j}$ and ${|g\rangle }_{j}$ denote the excited and ground states of the *j*-th emitter. Furthermore, the creation $a_{k}^{†}$ and annihilation $a_{k}$ operators describe the thermal reservoir of the environmental electromagnetic field (EMF). These bosonic operators obey the standard commutation relations $\left[a_{k},a_{k^{\prime }}^{†}\right]=\delta_{kk^{\prime }}$ and $\left[a_{k},a_{k^{\prime }}\right]$ = $[a_{k}^{†},a_{k^{\prime }}^{†}]=0$ [52].

In the interaction picture, the general master equation describing the dynamics of the atomic subsystem alone is given by [8,9]

(5) $$\begin{array}{c} \dot{\rho }\left(t\right)=-\frac{1}{\hbar^{2}}{\mathrm{Tr}}_{f}\left\{\int_{0}^{t}dt^{\prime }\left[H_{I}\left(t\right),\left[H_{I}\left(t^{\prime }\right),\rho \left(t^{\prime }\right)\right]\right]\right\}, \end{array}$$

where the interaction Hamiltonian $H_{I}\left(t\right)$ is defined as

(6) $$\begin{array}{c} H_{I}\left(t\right)=U\left(t\right)H_{AF}U^{-1}\left(t\right), \end{array}$$

with the unitary evolution operator $U\left(t\right)=e^{iH_{0}t/\hbar }$, while the overdot means differentiation with respect to time. The unperturbed Hamiltonian $H_{0}$ has the form

(7) $$\begin{array}{c} H_{0}=\sum_{k}\hbar \omega_{k}a_{k}^{†}a_{k}+\hbar {\bar{\omega }}_{0}R_{z}-\hbar \bar{\Omega }R^{+}R^{-}, \end{array}$$

whereas the renormalized frequency is ${\bar{\omega }}_{0}=\omega_{0}+\bar{\Omega }$. In deriving Equation (7), we used the relation $R^{+}R^{-}$=$\sum_{j\neq l}R_{j}^{+}R_{l}^{-}$ + $\sum_{j}R_{zj}+N/2$, where the constant term N/2 has been omitted. Furthermore, we account for the additive nature of the Hamiltonian describing multiple two-level radiators, which comprises a sum of individual Hamiltonians for each separate emitter. Consequently, the dipole–dipole coupling strength Ω is scaled by N−1, yielding $\bar{\Omega }=\Omega /\left(N-1\right)$, because there are N(N−1) terms representing the interacting atoms [6]. The static dipole–dipole interaction strength scales as $\Omega ∼d^{2}/r^{3}$ [6], where *d* is the dipole moment and *r* denotes the mean distance between any atomic pair. This coupling is assumed sufficiently strong, so the ratio $\Omega \omega_{0}^{-1}<1$ is non-negligible, potentially influencing the established thermal equilibrium features. We consider that the dipole–dipole coupling strength is identical for all atomic pairs, which serves as a reasonable approximation in the Dicke limit. Alternatively, one can assume a Gaussian-distributed atomic cloud to obtain an averaged dipole–dipole coupling strength Ω; see, e.g., Ref. [53]. Finally, the notation ${\mathrm{Tr}}_{f}\left\{\cdots \right\}$ denotes the partial trace over the degrees of freedom of the thermal EMF.

The explicit expression of the interaction Hamiltonian, in the interaction picture, is

(8) $$\begin{array}{c} H_{I}=i\sum_{k}\left({\vec{g}}_{k}\cdot \vec{d}\right)a_{k}^{†}e^{i(\omega_{k}-\hat{\omega })t}R^{-}+H.c., \end{array}$$

where $\hat{\omega }={\bar{\omega }}_{0}+2\bar{\Omega }R_{z}$. Next, by substituting the Hamiltonian (8) into Equation (5), we perform the partial trace over the thermal EMF degrees of freedom, while explicitly accounting for the operator nature of the exponential function. Subsequently, within the framework of the Born–Markov approximations, we derive the following master equation that describes the atomic subsystem alone [16]:

(9) $$\begin{array}{ccc} \dot{\rho }\left(t\right) & + & i\left[{\bar{\omega }}_{0}R_{z}-\bar{\Omega }R^{+}R^{-},\rho \right]=-\left[R^{+},\hat{\Gamma }\left(\hat{\omega }\right)\left(1+\bar{n}\left(\hat{\omega }\right)\right)R^{-}\rho \right]/2\\ & - & \left[R^{-},R^{+}\hat{\Gamma }\left(\hat{\omega }\right)\bar{n}\left(\hat{\omega }\right)\rho \right]/2+H.c.. \end{array}$$

Here, $\hat{\Gamma }\left(\hat{\omega }\right)=4d^{2}{\hat{\omega }}^{3}/\left(3\hbar c^{3}\right)$ represents the spontaneous decay rate of the emitters, whereas $\bar{n}\left(\hat{\omega }\right)={\left[\exp\left(\hbar \hat{\omega }/k_{B}T\right)-1\right]}^{-1}$ denotes the mean thermal photon number. Both quantities are evaluated at the eigenvalues of the operator $\hat{\omega }$. Although finding the general solution to the master Equation (9) is challenging for a many-atom system, a steady-state solution exists. Direct substitution into Equation (9) verifies that the density matrix

(10) $$\begin{array}{c} \rho_{s}=Z^{-1}\exp\left[-\beta H_{a}\right], \end{array}$$

is a steady-state solution [16]. Here, the normalization constant *Z* is determined by the condition $\mathrm{Tr}\{\rho_{s}\}=1$, and the effective Hamiltonian is given by $H_{a}=\hbar {\bar{\omega }}_{0}R_{z}-\hbar \bar{\Omega }R^{+}R^{-}$, where $\beta ={\left(k_{B}T\right)}^{-1}$ and $k_{B}$ is the Boltzmann constant.

In the next Section, we shall focus on the steady-state behaviors of the von Neumann entropy in dense atomic ensembles, whose thermodynamic equilibrium is described by the solution (10). Actually, the focus will be on comparing these behaviors when $\Omega /\omega_{0}\to 0$, a case which is mostly known in the scientific literature, with the situation considered here: namely, when $\Omega \omega_{0}^{-1}\neq 0$, while $\Omega /\omega_{0}<1$.

## 3. Results and Discussion

For a quantum system described by the density operator $\rho_{s}$, the von Neumann entropy is defined as

(11) $$S=-k_{B}Tr\left(\rho_{s}\ln\rho_{s}\right).$$

Since the von Neumann entropy depends only on the eigenvalues of the density operator, it is invariant under unitary transformations and thus provides a basis-independent measure of the mixedness of a quantum state, giving a vanishing value for a pure state and attaining its maximum value, S(ρ)=lnd, for the maximally mixed state ρ=I/d in a *d*-dimensional Hilbert space [54]. A few key mathematical properties of the von Neumann entropy clarify how entropy is shared across correlated subsystems, which are, respectively, concavity under statistical mixing, additivity for independent product states, and subadditivity [54]. For a globally pure bipartite state, the reduced states of the two subsystems have the same von Neumann entropy, which in this case provides a direct measure of their bipartite entanglement [21]. These features make the von Neumann entropy particularly useful for describing changes in the purity of open quantum systems and the redistribution of information and correlations among interacting subsystems.

Using the symmetric Dicke states |n〉—which denote symmetrized *N*-atom states with *n* excited atoms and N−n atoms in the ground state—along with the relations $R^{-}|n\rangle =\sqrt{n(N-n+1)}|n-1\rangle$, $R^{+}|n\rangle =\sqrt{\left(N-n)(n+1\right)}|n+1\rangle$ and $R_{z}|n\rangle =\left(n-N/2\right)|n\rangle$, one can evaluate the steady-state expectation value of any relevant atomic correlator [5,6,7,8,9,10]. Particularly, for a single-qubit system, i.e., {Ω=0,N=1}, the entropy is given as

(12) $$S=-k_{B}R_{gg}\ln R_{gg}-k_{B}R_{ee}\ln R_{ee},$$

with $R_{gg}=\left(1+\bar{n}\left(\omega_{0}\right)\right)/\left(1+2\bar{n}\left(\omega_{0}\right)\right)$ and $R_{ee}=\bar{n}\left(\omega_{0}\right)/\left(1+2\bar{n}\left(\omega_{0}\right)\right)$ being the single-atom populations of the ground and of the excited atomic states, respectively. A Dicke two-atom system, i.e., N=2, is characterized by the following expression for the entropy:

(13) $$S=-k_{B}R_{EE}\ln R_{EE}-k_{B}R_{SS}\ln R_{SS}-k_{B}R_{GG}\ln R_{GG},$$

where

(14) $$\begin{array}{ccc} R_{EE} & = & e^{-x_{-}-x_{+}}{\left(1+e^{-x_{-}}+e^{-x_{+}-x_{-}}\right)}^{-1},\\ R_{SS} & = & e^{-x_{-}}{\left(1+e^{-x_{-}}+e^{-x_{+}-x_{-}}\right)}^{-1},\\ R_{GG} & = & {\left(1+e^{-x_{-}}+e^{-x_{+}-x_{-}}\right)}^{-1}, \end{array}$$

are the populations of the two-atom Dicke states [16]: |E〉=|ee〉, $|S\rangle =(|eg\rangle +\left|ge\rangle \right)/\sqrt{2}$ and |G〉=|gg〉, respectively, with $x_{\pm }=\hbar \left(\omega_{0}\pm \Omega \right)/\left(k_{B}T\right)$. Thus, the final expression for the two-atom entropy is

(15) $$S=k_{B}\left[\ln\left(1+e^{x_{+}}+e^{x_{+}+x_{-}}\right)-\frac{e^{x_{+}}\left(x_{-}e^{x_{-}}+x_{+}\left(1+e^{x_{-}}\right)\right)}{1+e^{x_{+}}+e^{x_{+}+x_{-}}}\right],$$

and so on. Notice here that the sub-radiant (or antisymmetric) two-qubit state, i.e., $|A\rangle =(|eg\rangle -\left|ge\rangle \right)/\sqrt{2}$, was omitted since it decouples from the interaction with the environmental thermostat. Actually, the population time of the sub-radiant state is quite slow, proportional to $t∼{\left[\Gamma \left(\omega_{0}\right)\left(1-\chi \right)\right]}^{-1}$, where the collective contribution, i.e., χ, to the sub-radiant two-qubit decay rate is close to unity in the Dicke limit [9]. Therefore, the sub-radiant channels are not considered in our approach.

**Figure 1:**
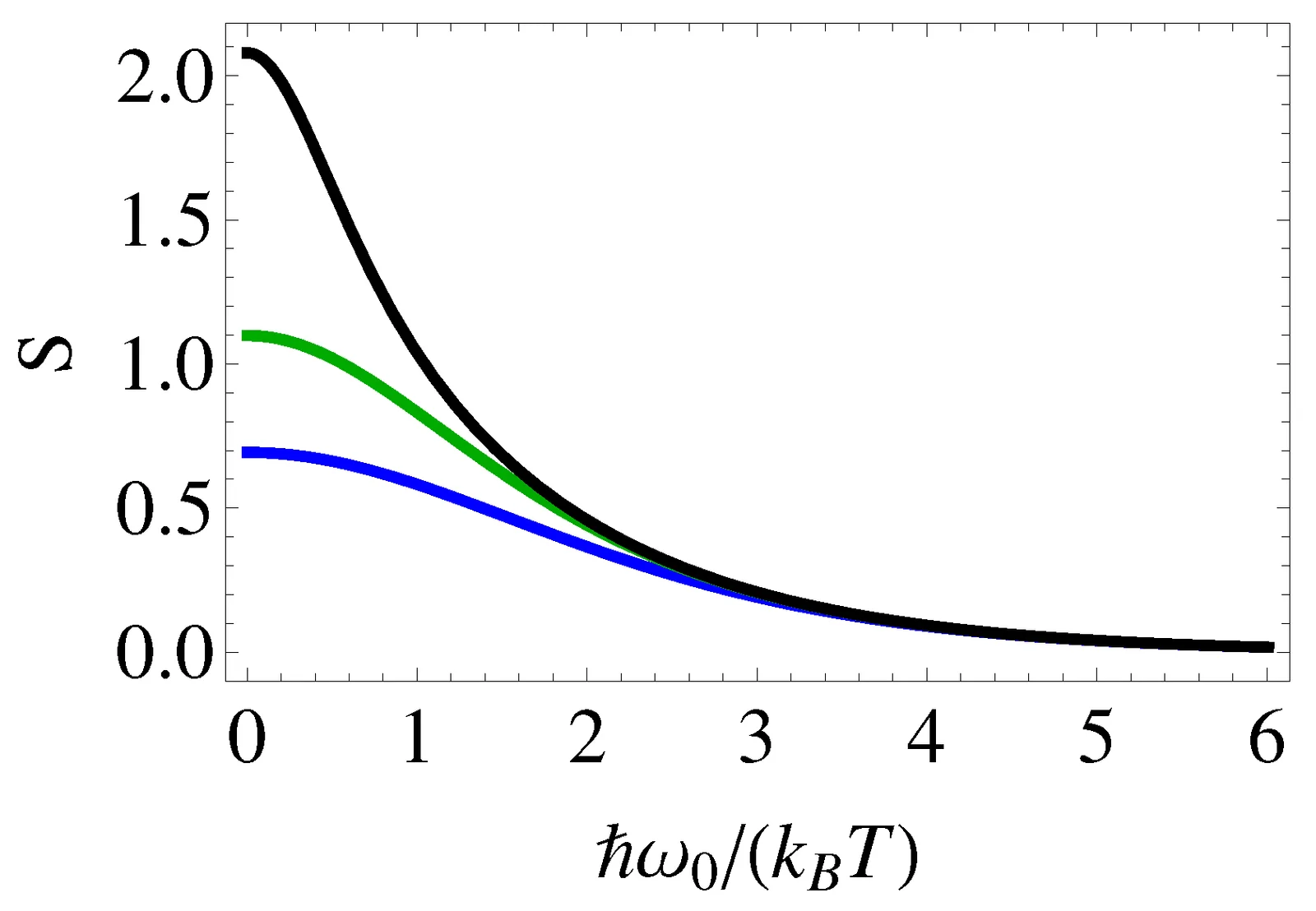
The von Neumann entropy S in units of $k_{B}$, against the ratio $\hbar \omega_{0}/k_{B}T$, in the Dicke limit and without considering the dipole–dipole interactions between the two-level emitters; that is, $\Omega /\omega_{0}=0$. Here, from bottom to top, N=1, N=2 and N=7, respectively.

**Figure 2:**
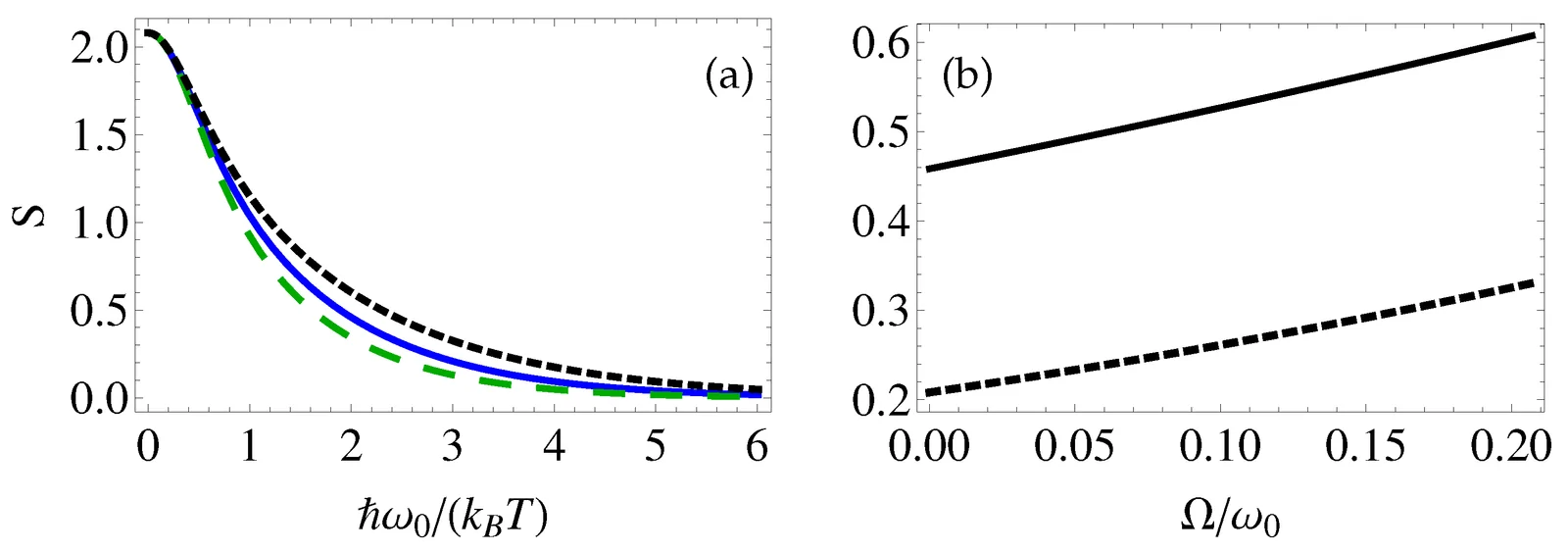
The collective von Neumann entropy S in units of $k_{B}$, for N=7, against the ratio (**a**) $\hbar \omega_{0}/k_{B}T$ (**b**) versus $\Omega /\omega_{0}$, respectively. (**a**) The solid blue line is plotted for $\Omega /\omega_{0}=0$, the long-dashed green curve is for $\Omega /\omega_{0}=-0.2$, and the black short-dashed one is for $\Omega /\omega_{0}=0.2$. (**b**) The solid black line depicts the case of $\hbar \omega_{0}/\left(k_{B}T\right)=2$, whereas the short-dashed one depicts that of $\hbar \omega_{0}/\left(k_{B}T\right)=3$.

Figure 1 depicts the von Neumann entropy for N=1, N=2 and N=7 atoms, without considering the dipole–dipole interactions among the emitters. On the other side, in Figure 2a, we fix the number of the emitters in the sample, i.e., N=7, and make plots with the dipole–dipole coupling strengths included. Generally, one can observe that the von Neumann entropy is a monotonically increasing function against the temperature, *T*. Regardless of the presence of dipole–dipole interactions within the Dicke two-level ensemble, it is maximal when $x\equiv \hbar \omega_{0}/\left(k_{B}T\right)\to 0$, namely, $S=k_{B}\ln\left(1+N\right)$. This is due to a final created mixed collective state, comprising half of the atoms being in their excited state and half in their ground state, respectively. Actually, all the symmetric collective Dicke states are equally populated in this particular case. Furthermore, S=0 when x≫1, meaning that all atoms reside in their ground state, which is a final pure state. However, in the presence of strong dipole–dipole interactions among the two-level emitters embedded in a thermal bath, the von Neumann entropy enhances with respect to the situation when the dipole–dipole couplings are negligibly small compared to the transition frequency $\omega_{0}$, if Ω has a positive sign. Conversely, it decreases for a negative sign of Ω; compare the long- and short-dashed curves in Figure 2a. Though we fixed here the number of atoms to N=7, similar behaviors occur for different and moderate values of *N*.

Figure 2b depicts the steady-state behaviors of the von Neumann entropy, *S*, against the positive ratio of the dipole–dipole coupling strength over the emitter’s transition frequency, $\Omega /\omega_{0}$, for $\hbar \omega_{0}/\left(k_{B}T\right)=2$ and $\hbar \omega_{0}/\left(k_{B}T\right)=3$, respectively, proving the entropy’s enhancement even for $\Omega /\omega_{0}\ll 1$. The physical mechanism for entropy enhancement is as follows: the dipole–dipole term lowers the excited collective multiplets relative to the collective ground state so that the first excitation gap is reduced while the population of the first excited collective state slightly increases because of the thermal environment, and hence the entropy increases as well. Similarly, the entropy decreases for an opposite sign of the dipole–dipole coupling strength, since the first excitation gap increases while the first cooperative state is getting correspondingly less populated. Finally, notice that since we considered small ratios of $\Omega /\omega_{0}<1$, the fully unexcited state remains the collective ground state.

## 4. Summary

We have investigated the von Neumann entropy for a dense Dicke-like atomic ensemble, consisting of motionless two-level emitters confined in a fully symmetric Dicke subspace and interacting via their dipole–dipole couplings when embedded in an environmental thermal reservoir. We present the analytical solution of the master equation, characterizing the atomic ensemble in the thermodynamic equilibrium, and describe the behaviors of the von Neumann entropy. As a result, we have demonstrated that the von Neumann entropy can be enhanced or reduced depending on the sign of the strong dipole–dipole couplings involved.

## Data Availability

The original contributions presented in this study are included in the article. Further inquiries can be directed to the corresponding author.
